# Establishing Indicators and an Analytic Method for Moisture Susceptibility and Rutting Resistance Evaluation Using a Hamburg Wheel Tracking Test

**DOI:** 10.3390/ma13153269

**Published:** 2020-07-23

**Authors:** Wei-Han Wang, Chien-Wei Huang

**Affiliations:** Department of Civil Engineering, National Chung Hsing University, Taichung 40227, Taiwan; a8505099@gmail.com

**Keywords:** Hamburg wheel tracking test, moisture susceptibility, rutting resistance, asphalt mixtures

## Abstract

The Hamburg wheel tracking test (HWTT) is widely used to evaluate the performance of asphalt mixtures. According to HWTT specifications, the stripping inflection point (SIP) and the rut depth at a certain number of load cycles are two common indicators for evaluating the moisture susceptibility and rutting resistance of asphalt mixtures, respectively. Although these indicators have been used extensively by several transportation institutions, the reliability and stability in evaluating asphalt mixture behaviors of these indicators have been questioned. To more effectively evaluate the performance of asphalt mixture in the HWTT, this study introduces a novel method of analysis for the HWTT and novel indicators of rutting resistance and moisture susceptibility. The proposed method and indicators were employed to analyze the HWTT results of 14 field core specimens, and the proposed indicators were compared with conventional HWTT indicators to assess their capability of distinction between asphalt mixtures with different performance behaviors in the HWTT. The results indicate that the conventional HWTT indicators cannot effectively evaluate the asphalt mixtures with different performance in the HWTT. By contrast, the proposed analytic method and indicators have significant advantages to effectively evaluate and distinguish the rutting resistance and moisture susceptibility of asphalt mixtures.

## 1. Introduction

The Hamburg wheel tracking test (HWTT) was developed by Esso A.G. in Hamburg, Germany [1]. The test involves immersing an asphalt concrete specimen in a water bath at a specific temperature, generally 50 °C, and then using a steel wheel for repeated loading to determine the relationship between the load cycle and rut depth. Specimens used in the HWTT can be either a slab specimen or cylindrical specimen. Slab specimens are generally fabricated in a laboratory with a slab compactor, whereas cylindrical specimens can be prepared using a Superpave Gyratory Compactor (SGC) in the laboratory or obtained from cores onsite [2].

The HWTT has been widely used in laboratories to evaluate the rutting resistance and moisture susceptibility of asphalt mixture. Numerous studies have assessed the effectiveness of various wheel tracking tests in evaluating the rutting resistance and moisture susceptibility of asphalt mixture. Han et al. [3] compared the effectiveness of the asphalt pavement analyzer (APA) test and HWTT in evaluating the susceptibility of asphalt mixture to moisture damage. The results revealed significant stripping of specimens in the APA tests; however, APA tests did not indicate any stripping inflection point (SIP), whereas the HWTT results did. Furthermore, the HWTT results indicated considerable improvement in the antistripping performance of the asphalt mixture by the addition of antistripping additives; therefore, the HWTT is more effective than the APA in determining the susceptibility of an asphalt mixture to moisture damage. Stuart et al. [4] used the HWTT, French pavement rutting tester, and Georgia loaded-wheel tester to evaluate the rutting resistance of an asphalt mixture mixed with different asphalt binder grades. The experimental results revealed that the Georgia loaded-wheel tester demonstrated a significant relationship between rutting and the rheological properties of the binders; the HWTT and French pavement rutting tester failed to yield the same result. In addition, a number of researchers employed the HWTT to evaluate the effects of aggregate properties, aggregate gradation, types of asphalt, short-term aging, and temperature on asphalt mixtures [5,6,7,8,9,10] and proposed different rutting resistance and moisture susceptibility indicators for the HWTT. Uppu et al. [11] used the number of wheel passes, creep slope, and SIP as indicators to evaluate the moisture susceptibility of reclaimed asphalt material with different asphalt binder contents. The research determined that the aforementioned HWTT indicators can indicate the moisture susceptibility of reclaimed asphalt material with different asphalt binder contents. Kim et al. [12] used the number of loading passes at failure, rut depth at 20,000 wheel passes, SIP, creep slope, and stripping slope in the HWTT to evaluate the rutting resistance and moisture susceptibility of the asphalt mixture. The results revealed that the number of loading passes at failure, rut depth at 20,000 wheel passes, and SIP are related to the nominal maximum aggregate size (NMAS) and that the creep slope is not significantly related to NMAS. Larrain et al. [13,14] used rut depth and the maximum number of load cycles in the HWTT as indicators to evaluate the effects of binder grade, test temperature, aggregate gradation, and aggregate types on the rutting resistance of asphalt mixtures. The results suggested that test temperature, type of aggregate, and aggregate gradation significantly influenced the aforementioned indicators. Schram et al. [15] proposed using HWTT results (i.e., SIP, creep slope, stripping slope, and the ratio between the stripping slope and creep slope) to evaluate the moisture susceptibility of asphalt mixture with different binder grades and NMAS and compared them with field performance observations. Izzo et al. [6] adopted creep slope, stripping slope, and SIP indicators to evaluate the moisture susceptibility of asphalt mixtures mixed with antistripping additives. Walubita et al. [16] proposed analyzing the results of the HWTT by using the shape factor, rutting area, and normalized rutting area to evaluate the rutting resistance of asphalt mixture; this research also compared the newly formulated and conventional parameters with field observation results.

Multiple researchers have proposed methods for the analysis of HWTT data. Al-Khateeb et al. [17] analyzed HWTT results by using a third-order-type three-stage model to determine the number of load cycles at which the three phases of the HWTT curve (post-compaction, creep, and stripping phase) change; the objective of the study was to evaluate the rutting resistance of asphalt mixtures and compare them with the rutting indicators of asphalt binders. Tsai et al. [18] suggested using the three-stage Weibull model to analyze the fatigue damage behavior of asphalt mixtures and subsequently applied the three-stage Weibull model to analyze the HWTT rutting curve [19]. The study indicated that the model can improve existing HWTT rutting curve evaluation methods and provide a systematic analysis for SIP evaluation. Yin et al. [20] used a viscoplastic model to fit the HWTT rutting curve of asphalt mixtures that had not been damaged by moisture. The research proposed using the curve of the viscoplastic model to evaluate the viscoplastic strain of asphalt mixtures at a given number of load cycles (as an indicator of rutting resistance). Yin et al. then extended the viscoplastic curve to propose the number of load cycles (indicating moisture susceptibility) required when rutting in the HWTT reaches a particular value.

Although many transportation agencies have extensively used the HWTT to evaluate the rutting resistance and moisture susceptibility of asphalt mixtures, a number of problems remain in relation to data analysis based on HWTT specifications and the reliability and stability of rutting resistance and moisture susceptibility indicators [19,20,21]. For example, the HWTT rutting curve can be divided into three main phases, namely, post-compaction, creep, and stripping phases; each phase has its own mechanical behaviors and mechanisms [19,20]. Furthermore, the HWTT rutting curve includes permanent deformation due to moisture damage and due to repeated loading. Hence, at a given load cycle, rut depth cannot be used effectively to evaluate the resistance of the asphalt mixture to rutting [20]. In addition, a specimen reaches a failure state at a later HWTT stage, when its aggregate already displays obvious signs of stripping, which causes the aggregate to fall onto the wheel path. This condition leads to vibration of the wheel, creating instability in the data of the later stage of the HWTT, which affects the calculation of the SIP [21]. Therefore, establishing a method for systematic analysis and proposing novel indicators are necessary for effective use of the HWTT to evaluate the moisture susceptibility and rutting resistance of the asphalt mixture.

## 2. Objectives

The objective of this study was to develop a novel method for analyzing HWTT results. Two new indicators were introduced to evaluate the rutting resistance and moisture susceptibility of asphalt mixtures. In the study, the HWTT was conducted on 14 field core specimens. The results were analyzed using both the current HWTT protocol and the proposed method. Finally, the proposed and conventional HWTT indicators were compared to demonstrate their effectiveness in evaluating the rutting resistance and moisture susceptibility of asphalt mixtures with different performance behaviors in the HWTT.

## 3. Specimen Preparation and the HWTT

**Figure 1:**
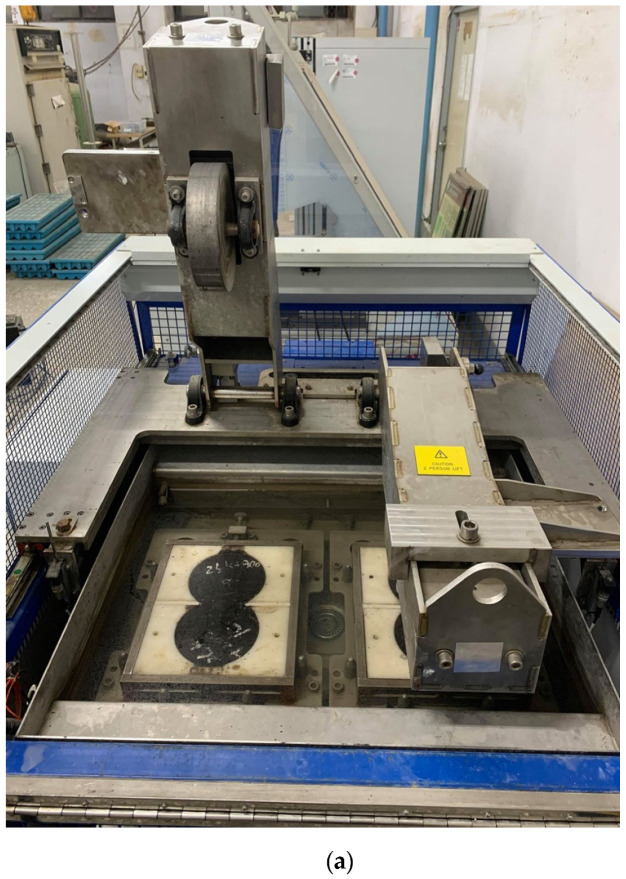

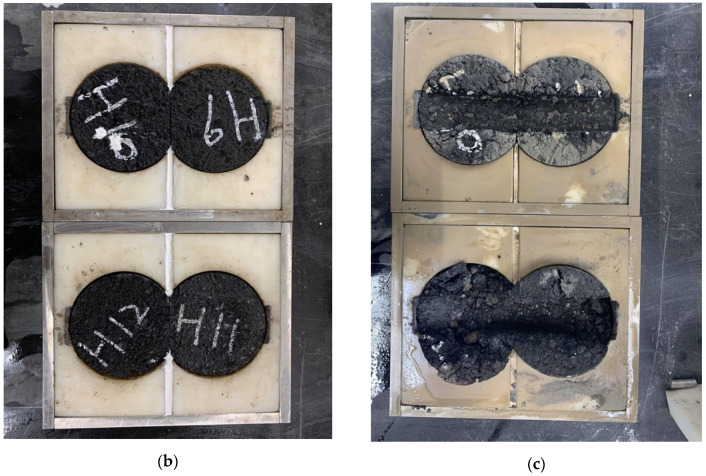
HWTT device and specimens. (**a**) HWTT device; (**b**) before test; (**c**) after test.

Field core specimens were prepared and tested following the AASHTO T324-17 HWTT specification (AASHTO, Washington, DC, USA) [2]. Figure 1a shows the HWTT device (Cooper, Ripley, UK). In the HWTT, a steel wheel with a diameter of 203.2 ± 2.0 mm and a width of 47 mm was applied at a frequency of 52 ± 2 passes per min. The load of the wheel is 705 ± 4.5 N. The wheel reciprocates over the specimen and the position of the wheel varies sinusoidally over time. The maximum speed of the wheel is approximately 0.305 m/s at the midpoint of the specimen.

The core specimens of asphalt mixture (Taichung, Taiwan) adopted in this study were 150 mm in diameter and thicker than 40 mm. According to HWTT specifications, the field core cylindrical specimens were sawed to a thickness of 40 mm. Two specimens were then sawed along equal secant lines and joined together along the cut edge in a mold, as shown in Figure 1b. Plaster (Chung Fan Gypsum CO., LTD., Taipei, Taiwan) was poured to fill the gap between the sides of the mold and specimen and allowed at least 1 h to set. Finally, the prepared specimens were immersed in a water bath (Cooper, Ripley, UK) at 50 °C for 30 min before the test commenced. Figure 1c shows the HWTT specimens after testing.

**Figure 2:**
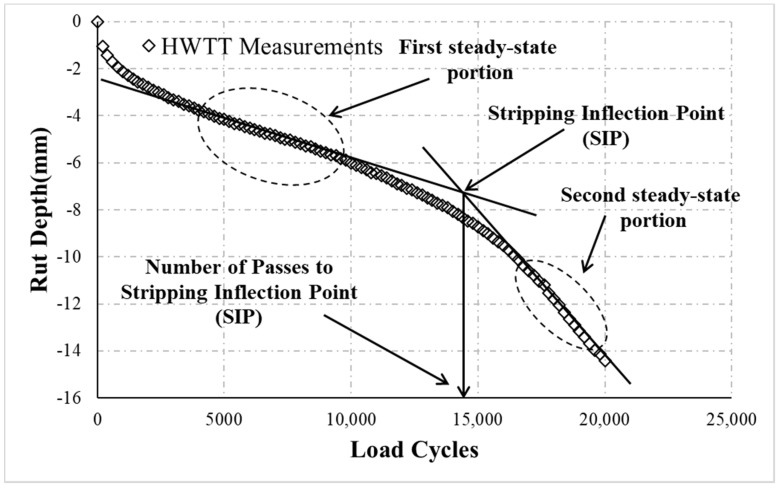
Relationship between the HWTT rut depth and load cycles.

The HWTT records the relationship between rut depth and load cycle as shown in Figure 2. According to HWTT specifications, the rutting resistance of asphalt mixtures is evaluated by the rut depth at a certain number of load cycles [2]. A greater rut depth indicates that the asphalt mixture has a lower resistance to rutting. In addition, HWTT specifications use the SIP to evaluate the moisture susceptibility of asphalt mixtures [2]. The SIP is defined as the number of load cycles at the point of intersection between two fitted straight lines from the first and second steady-state portions of the HWTT curve (dashed line with circles in Figure 2). The SIP is described by Equation (1):

(1) $$\mathrm{SIP}=\frac{b^{2}-b^{1}}{s^{1}-s^{2}}$$

where b and s are the interception and slope of the fitted straight lines, respectively. The superscript of b and s denotes the first and second steady-state portions of the curve. A high SIP indicates that the asphalt mixture has robust resistance to moisture damage.

## 4. Data Analysis Methodology

**Figure 3:**
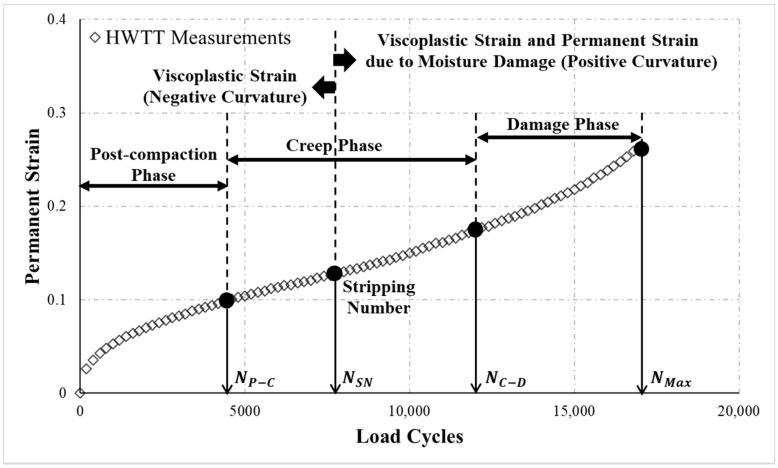
A schematic of the relationship between permanent strain and load cycles in the HWTT.

First, the rut depth obtained from the HWTT (Figure 2) was converted into permanent strain by using Equation (2). Figure 3 portrays the relationship between the calculated permanent strain and load cycles:

(2) $$\varepsilon_{p}=\frac{\left|{\mathrm{RD}}_{N}\right|}{T}$$

where ${\mathrm{RD}}_{N}$ is the rut depth (mm) at the N^th^ load cycle, T is the specimen thickness (mm), and $\varepsilon_{p}$ is the permanent strain.

### 4.1. Determination of the Critical Loading Number in HWTT

Since the HWTT applies repeated loading on a specimen in the presence of water, the total permanent strain in the HWTT simultaneously contains two components: viscoplastic strain (rutting) of the asphalt mixture itself due to repeated loading and permanent strain by the moisture effect (moisture damage). However, rutting resistance and moisture susceptibility of asphalt mixtures have different mechanical behaviors and mechanisms. Specifically, the rutting resistance of the asphalt mixture is more related to the distribution of aggregate in the asphalt mixture and to the properties of asphalt binders (e.g., aggregate gradation, aggregate property, and binder grade), whereas moisture susceptibility is affected by the surface properties of aggregate and bonding force of asphalt binders (e.g., hydrophilic or hydrophobic properties of the aggregate, and bonding energy between the aggregate and binder) [20].

Moreover, the permanent strain of asphalt mixture in the HWTT can be divided into three phases: (a) post-compaction phase, (b) creep phase, and (c) damage phase, as shown in Figure 3. The permanent strain in the post-compaction phase is mainly contributed by the consolidation of the specimen that occurs as the repeated loading densifies the mixtures and the air voids significantly decrease, causing permanent strain to increase as the load cycle increases. In the creep phase, the permanent strain is caused by viscous flow of the asphalt mixture and increases at an approximately constant rate with increasing load cycles. In the damage phase, the asphalt mixture is subjected to substantial moisture damage resulting in considerable loss of bonding between the asphalt binder and the aggregate, which causes visible damage to the asphalt mixture, such as stripping or raveling, and permanent strain to increase drastically with additional load cycles [22].

The behaviors and mechanisms at each phase of the HWTT curve thus differ completely. Therefore, to effectively use the HWTT for evaluating the rutting resistance and moisture susceptibility of asphalt mixture, it is necessary to determine the load cycle at which moisture damage to the asphalt mixture begins and the transition loading number for the three phases of the curve.

#### 4.1.1. Determination of the Stripping Number $N_{\mathrm{SN}}$

To determine the number of load cycles at which moisture damage to asphalt mixture begins in the HWTT, the Francken model [23], presented in Equation (3), was adopted to fit the curve of the relationship between the calculated permanent strain and the number of load cycles:

(3) $$\varepsilon_{p}=a_{1}N^{a_{2}}+a_{3}\left[\exp\left(a_{4}\times N\right)-1\right]$$

where N is the number of load cycles; $a_{1},{\;a}_{2},{\;a}_{3},{\;\mathrm{and}\;a}_{4}$ are the Francken model parameters. The Francken model parameters can be obtained by fitting the HWTT measurements.

The Francken model consists of two parts: a negative curvature followed by a positive curvature, as illustrated in Figure 3. A negative curvature means that the asphalt mixture hardens by repeated loading; here, permanent strain is provided mainly by the viscoplastic strain of the asphalt mixture itself. Therefore, this part of the curve can be used to evaluate the rutting resistance of asphalt mixture in the presence of water. In the part of the curve with positive curvature, the asphalt mixture is affected by water penetration, which weakens the bond between the aggregate and the asphalt binder, causing a reduction of the strength of the asphalt mixture. The behaviors then are related not only to the viscoplastic strain of the asphalt mixture itself but also to the effect of moisture damage on the asphalt mixture. Hence, the curve here can be used to evaluate the moisture susceptibility of the asphalt mixture [20,24]. In sum, the number of load cycles required for the HWTT permanent strain curvature to change from negative to positive can be considered the number of load cycles at which moisture damage to the asphalt mixture begins to occur (stripping number, $N_{\mathrm{SN}}$). In other words, the permanent strain of asphalt mixture before $N_{\mathrm{SN}}$ is mainly provided by the viscoplastic strain of the material itself, whereas the permanent strain after $N_{\mathrm{SN}}$ is contributed simultaneously by viscoplastic strain and permanent strain induced by moisture damage.

The curvature of the Francken model can be obtained by the second derivative of Equation (3). To determine $N_{\mathrm{SN}}$, the curvature of the Francken model as shown in Equation (4) is set to zero.

(4) $$\frac{\partial^{2}\varepsilon_{p}}{\partial N^{2}}=a_{1}a_{2}\left(a_{2}-1\right)N^{a_{2}-2}+a_{3}a_{4}^{2}\exp\left(a_{4}\times N\right)$$

#### 4.1.2. Determination of the Transition Loading Number between Post-Compaction and Creep Phase $N_{P-C}$

To determine the transition loading number in the three phases of the HWTT curve, a three-stage model [22] was adopted to analyze the HWTT measurements. According to the experimental measurements, the permanent strain before $N_{\mathrm{SN}}$ contained both the post-compaction phase and creep phase, as shown in Figure 3. Therefore, the permanent strain before $N_{\mathrm{SN}}$ was first analyzed to determine the transition loading number $N_{P-C}$, at which point the post-compaction phase transitions to the creep phase. The three-stage model expresses the permanent strain in the post-compaction phase and the creep phase by the power law and linear model, respectively, as described in Equations (5) and (6):

(5) $$\varepsilon_{p}^{\mathrm{st}1}=c_{1}N^{c_{2}}\;\;\;\;\;N\leq N_{P-C}$$

(6) $$\varepsilon_{p}^{\mathrm{st}2}=\varepsilon_{p}^{\mathrm{PS}}+c_{3}\left(N-N_{P-C}\right)\;{\;N}_{P-C}\leq N\leq N_{\mathrm{SN}}$$

where $\varepsilon_{p}^{\mathrm{st}1}$ and $\varepsilon_{p}^{\mathrm{st}2}$ are the permanent strain in the post-compaction phase and creep phase, respectively. $\varepsilon_{p}^{\mathrm{PS}}$ is permanent strain at the end of the post-compaction phase. $c_{1},{\;c}_{2}$ and $c_{3}$ are model parameters.

Since the HWTT curve is continuous, Equations (5) and (6) demonstrate that when$\;N=N_{P-C}$, $\varepsilon_{p}^{\mathrm{st}1}=\varepsilon_{p}^{\mathrm{st}2}=\varepsilon_{p}^{\mathrm{PS}}=c_{1}{\left(N_{P-C}\right)}^{c_{2}}$. In addition, the point at which the post-compaction phase intersects the creep phase ($N=N_{P-C}$) must also satisfy the continuity condition that slopes are equal, as expressed in Equation (7):

(7) $$\frac{\partial \varepsilon_{p}^{\mathrm{st}1}}{\partial N}=\frac{\partial \varepsilon_{p}^{\mathrm{st}2}}{\partial N}\;\;\mathrm{when}\;N=N_{P-C}$$

In Equation (7), the first derivative of $\varepsilon_{p}^{\mathrm{st}1}$ and $\varepsilon_{p}^{\mathrm{st}2}$ is expressed as Equations (8) and (9), respectively.

(8) $$\frac{\partial \varepsilon_{p}^{\mathrm{st}1}}{\partial N}=c_{1}c_{2}N^{c_{2}-1}$$

(9) $$\frac{\partial \varepsilon_{p}^{\mathrm{st}2}}{\partial N}=c_{3}$$

Equations (8) and (9) are substituted in Equation (7), obtaining $N_{P-C}$ as in Equation (10):

(10) $$N_{P-C}={\left(\frac{c_{3}}{c_{1}c_{2}}\right)}^{\frac{1}{c_{2}-1}}$$

Equations (5), (6), and (10) were used to analyze permanent strain before $N_{\mathrm{SN}}.$ First, an initial $N_{P-C}^{\mathrm{ini}}$ was assumed, and let $N_{P-C}^{\mathrm{ini}}=N_{P-C}^{i}$. Then, Equations (5) and (6) were used to fit permanent strain before $N_{P-C}^{i}$ and the permanent strain between $N_{P-C}^{i}$ and $N_{\mathrm{SN}}$, respectively. Nonlinear regression analysis was conducted to modify the model parameters (i.e., $c_{1},{\;c}_{2}$, and $c_{3}$) so that errors of the model-derived permanent strain (Equations (5) and (6)) and the HWTT-derived permanent strain were minimized. The error was calculated using Equation (11). Next, the obtained model parameters (i.e., $c_{1},{\;c}_{2}$, and $c_{3}$) were substituted into Equation (10) to calculate $N_{P-C}^{i+1}$. If $N_{P-C}^{i+1}=N_{P-C}^{i}$, $N_{P-C}^{i}$ at this point is the transition loading number $N_{P-C}$ between the post-compaction and creep phases.

(11) $${\mathrm{Err}}_{1}=\overset{N_{P-C}}{\underset{k=1}{\sum }}\left|\frac{\left(\varepsilon_{p}^{\exp,k}-\varepsilon_{p}^{\mathrm{model},k}\right)}{\varepsilon_{p}^{\exp,k}}\right|+\overset{N_{\mathrm{SN}}}{\underset{k=N_{P-C}}{\sum }}\left|\frac{\left(\varepsilon_{p}^{\exp,k}-\varepsilon_{p}^{\mathrm{model},k}\right)}{\varepsilon_{p}^{\exp,k}}\right|$$

where ${\mathrm{Err}}_{1}$ is error of HWTT-derived and model-derived permanent strain before $N_{\mathrm{SN}}$. $\varepsilon_{p}^{\exp,k}$ is permanent strain at the k^th^ point in the HWTT. $\varepsilon_{p}^{\mathrm{model},k}$ is permanent strain at the k^th^ point calculated using the model (Equations (5) or (6)). The algorithm is summarized as follows.

1. Assume an initial $N_{P-C}^{\mathrm{ini}}$ and set $N_{P-C}^{\mathrm{ini}}=N_{P-C}^{i}$.
2. Use Equations (5) and (6) to fit the experimental permanent strain before $N_{P-C}^{i}$ and between $N_{P-C}^{i}$ and $N_{\mathrm{SN}}$, respectively.
3. Obtain the model parameters $c_{1},{\;c}_{2},{\;\mathrm{and}\;c}_{3}\;$by minimizing the error function (Equation (11)).
4. Calculate $N_{P-C}^{i+1}$ (Equation (10)) using the model parameters $c_{1},{\;c}_{2},{\;\mathrm{and}\;c}_{3}\;$ obtained in step 3.
5. Compare $N_{P-C}^{i+1}$ and $N_{P-C}^{i}$; if $N_{P-C}^{i+1}=N_{P-C}^{i}$, then $N_{P-C}^{i}$ is the desired $N_{P-C}$. Otherwise, set $N_{P-C}^{i+1}$ as the new transition loading number between the post-compaction and creep phases and return to step 2.

#### 4.1.3. Determination of the Transition Loading Number between Creep and Damage Phases $N_{C-D}$

Once the transition loading number $N_{P-C}$ was determined, permanent strain after $N_{P-C}$ was analyzed using the creep and damage phases of the three-stage model to determine the transition loading number at which the material transitions from the creep phase to the damage phase ($N_{C-D}$). The permanent strain in the damage phase of the three-stage model is described by Equation (12):

(12) $$\varepsilon_{p}^{\mathrm{st}3}=\varepsilon_{p}^{\mathrm{CP}}+c_{4}\times N_{C-D}\left\{\exp\left[c_{5}\times \left(N-N_{C-D}\right)\right]-1\right\}\;\;{\;N}_{C-D}\leq N_{\max}$$

where $\varepsilon_{p}^{\mathrm{st}3}$ is permanent strain in the damage phase and $\varepsilon_{p}^{\mathrm{CP}}$ is permanent strain at the end of the creep phase. $N_{\max}$ is the maximum loading number in the HWTT. $c_{4}$ and $c_{5}$ are model parameters.

Since the HWTT curve is continuous, Equations (6) and (12) reveal that when$\;N=N_{C-D}$, $\varepsilon_{p}^{\mathrm{st}2}=\varepsilon_{p}^{\mathrm{st}3}=\varepsilon_{p}^{\mathrm{CP}}=\varepsilon_{p}^{\mathrm{PS}}+c_{3}\left(N_{C-D}-N_{P-C}\right)$, and the point at which the creep phase intersects the damage phase ($N=N_{C-D}$) must also satisfy the following continuity condition:

(13) $$\frac{\partial \varepsilon_{p}^{\mathrm{st}2}}{\partial N}=\frac{\partial \varepsilon_{p}^{\mathrm{st}3}}{\partial N}\;\;\;\mathrm{when}\;N=N_{C-D}$$

The first derivative of $\varepsilon_{p}^{\mathrm{st}3}$ can be derived using Equation (12), as in Equation (14):

(14) $$\frac{\partial \varepsilon_{p}^{\mathrm{st}3}}{\partial N}=c_{4}\times c_{5}\times N_{C-D}\exp\left(N-N_{C-D}\right)$$

Equations (9) and (14) may be substituted into Equation (13), yielding $N_{C-D}$ as expressed in Equation (15):

(15) $$N_{C-D}=\frac{c_{3}}{c_{4}\times c_{5}}$$

The transition loading number at which the material transitions from the creep phase to the damage phase was determined by using Equations (6), (12), and (15) to analyze permanent strain after $N_{P-C}$. Moreover, the analysis range in Equation (6) was modified between $N_{P-C}$ and $N_{C-D}$ for creep phase. The analysis algorithm was similar to that for determining $N_{P-C}$. Since $c_{3}$ in Equation (15) was obtained during the iteration of $N_{P-C}$, only $c_{4}$ and $c_{5}$ had to be calculated for Equation (15). An initial $N_{C-D}^{\mathrm{ini}}$ was assumed, and $N_{C-D}^{\mathrm{ini}}=N_{C-D}^{i}$ was set. Then, Equation (6) was used to calculate the permanent strain between $N_{P-C}$ and $N_{C-D}^{i}$, and Equation (12) was used to calculate the permanent strain between $N_{C-D}^{i}\;$and $N_{\max}$. The model parameters (i.e., $c_{4}$ and $c_{5}$) were modified so that errors of the model- and HWTT-derived permanent strains were minimized. The error was calculated using Equation (16). Next, the obtained model parameters (i.e., $c_{4}$ and $c_{5}$) were substituted into Equation (15) to calculate $N_{C-D}^{i+1}$. If $N_{C-D}^{i+1}=N_{C-D}^{i}$, then $N_{C-D}^{i}$ at this point is the desired transition loading number $N_{C-D}$.

(16) $${\mathrm{Err}}_{2}=\overset{N_{C-D}}{\underset{k=N_{P-C}}{\sum }}\left|\frac{\left(\varepsilon_{p}^{\exp,k}-\varepsilon_{p}^{\mathrm{model},k}\right)}{\varepsilon_{p}^{\exp,k}}\right|+\overset{N_{\max}}{\underset{k=N_{C-D}}{\sum }}\left|\frac{\left(\varepsilon_{p}^{\exp,k}-\varepsilon_{p}^{\mathrm{model},k}\right)}{\varepsilon_{p}^{\exp,k}}\right|$$

where ${\mathrm{Err}}_{2}$ is accumulated error of the HWTT-derived and the model-derived permanent strain between $N_{P-C}$ and $N_{\max}$.

### 4.2. Rutting Resistance Evaluation

**Figure 4:**
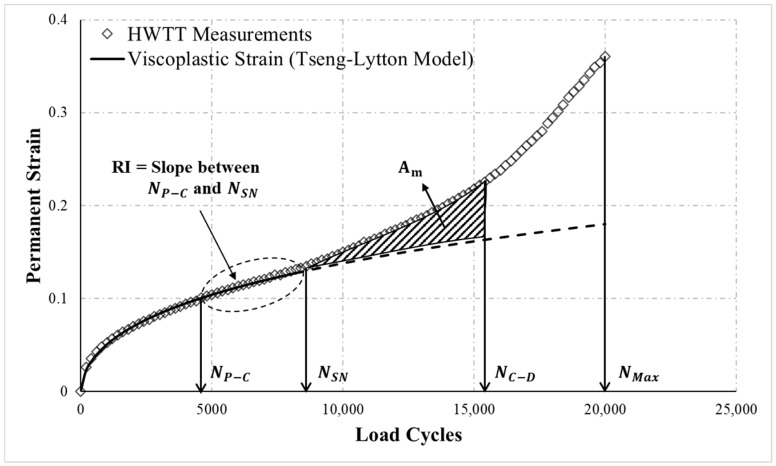
A schematic of RI and $D_{m}$ in the HWTT.

Since permanent strain before $N_{\mathrm{SN}}\;$was provided mainly by the permanent strain of the asphalt mixture itself under repeated loading, the data before $N_{\mathrm{SN}}$ was used to evaluate the rutting resistance of asphalt mixture. In addition, the permanent strain in the post-compaction phase is mainly due to the initial air void consolidation and not related to the rutting resistance of the asphalt mixture. Therefore, to eliminate the effect of the initial air void consolidation, the rutting resistance of the asphalt mixture was evaluated using the rate of change in permanent strain between$\;{\;N}_{P-C}$ and $N_{\mathrm{SN}}$ as shown in Figure 4. A rutting resistance index RI was proposed in this study as shown in Equation (17). The physical meaning of RI is the increased permanent strain of asphalt mixture at unit number of load cycle; higher RI means weaker resistance to rutting:

(17) $$\mathrm{RI}=\frac{\varepsilon_{p}^{N_{\mathrm{SN}}}-\varepsilon_{p}^{N_{C-D}}}{N_{\mathrm{SN}}-N_{C-D}}$$

where $\varepsilon_{p}^{N_{\mathrm{SN}}}$ and $\varepsilon_{p}^{N_{C-D}}$ are permanent strain at $N_{\mathrm{SN}}$ and $N_{C-D}$, respectively.

### 4.3. Moisture Susceptibility Evaluation

Permanent strain after $N_{\mathrm{SN}}$ is provided simultaneously by the permanent strain of asphalt mixture itself (viscoplastic strain) and permanent strain induced by moisture damage. Therefore, to effectively evaluate the moisture susceptibility of asphalt mixture, the permanent strain caused by moisture damage has to be extracted from HWTT measurements. This study employed the Tseng–Lytton model, a viscoplastic model has been commonly used in asphalt mixture, to fit the permanent strain before $N_{\mathrm{SN}}$ for representing the viscoplastic strain of asphalt mixture. The Tseng-Lytton model [25] is described by Equation (18). Using nonlinear regression analysis, the parameters of the Tseng-Lytton model (i.e., α, λ, and $\varepsilon_{\infty }^{\mathrm{vp}}$) were modified such that the error of model- and test-derived permanent strain was minimized:

(18) $$\varepsilon^{\mathrm{vp}}={\;\varepsilon }_{\infty }^{\mathrm{vp}}\exp{\left[-\left(\frac{\alpha }{N}\right)\right]}^{\lambda }$$

where $\varepsilon^{\mathrm{vp}}$ is viscoplastic strain and $\varepsilon_{\infty }^{\mathrm{vp}}$ is saturated viscoplastic strain. α and λ are model coefficients.

**Figure 5:**
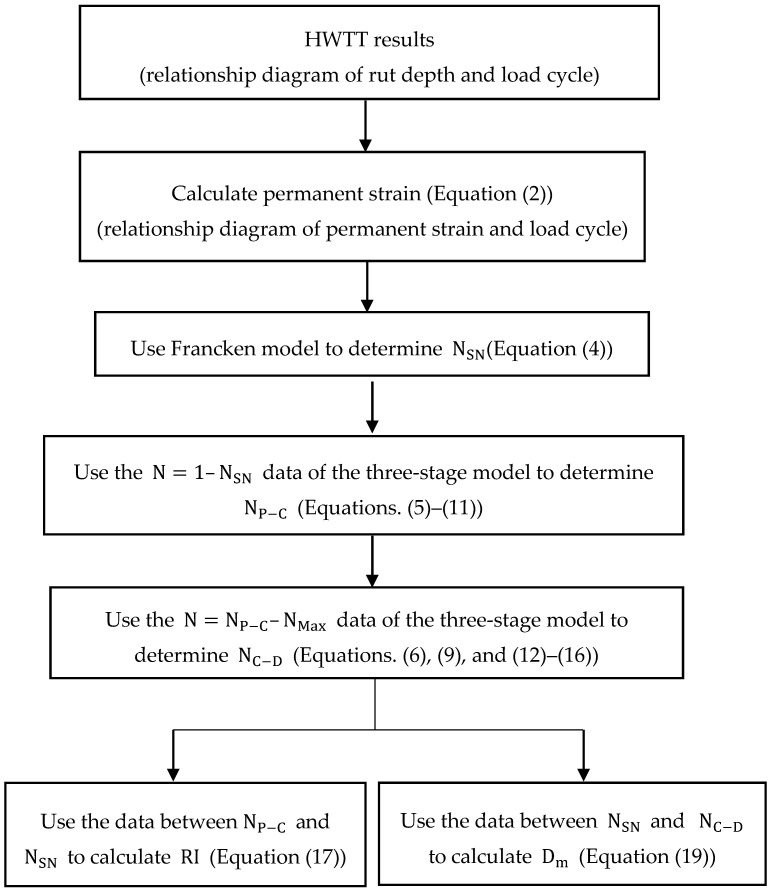
Flowchart of the analysis of RI and $D_{m}$ in the HWTT.

The viscoplastic strain curve was then extended by substituting the obtained parameters into the Tseng–Lytton model to determine viscoplastic strain after $N_{\mathrm{SN}}$, indicated by the dashed line in Figure 4. This extended curve represents the viscoplastic strain in the asphalt mixture generated after $N_{\mathrm{SN}}$ due to repeated loading; therefore, the effects of moisture damage on the asphalt mixture can be evaluated by the HWTT-based permanent strain and the Tseng–Lytton model-extended viscoplastic curve. However, the curve of HWTT after $N_{C-D}$ (damage phase) is often susceptible to serious moisture damage and therefore exhibits unstable conditions. To eliminate the effects of data instability after the failure of the specimen, data within the interval between $N_{\mathrm{SN}}$ and $N_{C-D}$ were adopted to evaluate moisture susceptibility. First, the area ($A_{m}$) between the curve of the permanent strain from HWTT and the Tseng–Lytton model-extended curve was calculated as shown in Figure 4. Second, this area was divided by the number of load cycles required between $N_{\mathrm{SN}}$ and $N_{C-D}$, the product of which was defined as $D_{m}$, an indicator of moisture susceptibility as shown in Equation (19). $D_{m}$ represents the effect of a unit load of moisture damage on asphalt mixture. The susceptibility of asphalt mixture to moisture damage increases with $D_{m}$. A flowchart of the proposed HWTT analytic method is provided in Figure 5. The analysis has been written into an automatic analysis program using Visual Basic in Excel (Microsoft, Redmond, Washington, DC, USA) to facilitate subsequent analyses.

(19) $$D_{m}=\frac{A_{m}}{N_{C-D}-N_{\mathrm{SN}}}$$

## 5. Results and Discussion

**Figure 6:**
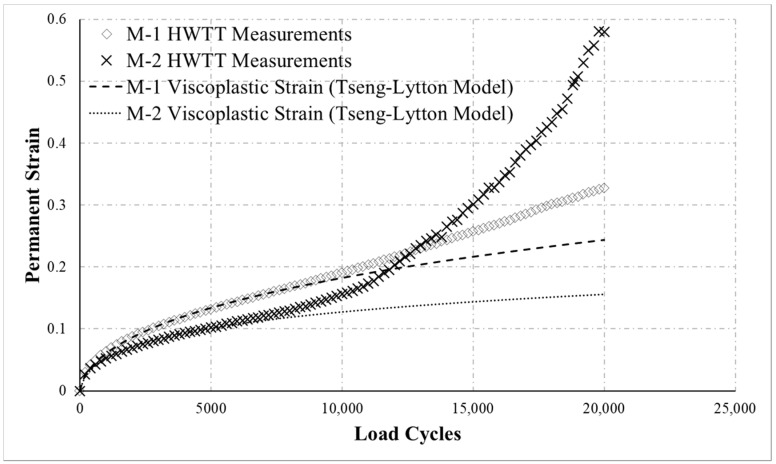
HWTT results for M-1 and M-2.

In this study, the HWTT was performed on 14 field core specimens (i.e., M-1, M-2, … M-14). The results were analyzed using the proposed novel analytic method to obtain RI and $D_{m}$, which were then compared with conventional HWTT indicators. Figure 6 presents the HWTT results of the M-1 and M-2 specimens. The results show that M-1 before 13,000 load cycles had larger permanent strain, whereas the opposite trend was observed as load cycle increased. The shape of the M-2 permanent strain curve in Figure 6 exhibits distinct post-compaction, creep and damage phases. Thus, M-2 was seriously damaged by moisture before 20,000 load cycles were completed, causing the permanent strain of M-2 to increase rapidly at the later stage of the test. Table 1 presents the permanent strain at different load cycles (i.e., 5000, 10,000, 15,000, and 20,000 cycles). As the table suggests, according to the definition of rutting resistance in HWTT specification, M-2 was more resistant to rutting at 5000 and 10,000 load cycles, but M-1 was more resistant at 15,000 and 20,000 load cycles. These results reveal that using rut depth at a certain number of load cycles to evaluate the rutting resistance of asphalt mixtures yields results that are significantly affected by the number of load cycles selected. Hence, the rutting resistance defined in HWTT specification does not adequately represent the accumulative permanent deformation behavior of the asphalt mixture.

As the curves in Figure 6 indicate, the permanent strain of M-1 in the creep phase accumulated significantly faster than that of M-2, indicating that M-1 was weaker than M-2 with regard to rutting resistance. The RI calculated following the flowchart of the method proposed in this study was 1.01 × 10^−5^ for M-1 and 8.11 × 10^−6^ for M-2 as shown in Table 1, which indicates that M-2 was more resistant to rutting, a result that fits with the curves in Figure 6. This analysis suggests that conventional HWTT indicators cannot effectively evaluate the permanent strain behavior of asphalt mixtures under repeated loading. The proposed analysis method and RI can more effectively evaluate and distinguish the rutting resistance of asphalt mixture with different rutting behaviors in the HWTT.

This study further analyzed the rutting resistance of M-1 and M-2 using the method proposed by Yin et al. [20]. This method also employed the viscoplastic model to evaluate the rutting resistance of asphalt mixtures. The rutting resistance index $\Delta \varepsilon_{10,000}^{vp}$ was characterized by the viscoplastic strain increment at 10,000 load cycles [20]. The $\Delta \varepsilon_{10,000}^{vp}$ was 7.82 × 10^−6^ for M-1 and 4.57 × 10^−6^ for M-2, as shown in Table 1, which indicates that M-2 was more resistant to rutting. This result was consistent with the proposed RI results, revealing that it is necessary to effectively evaluate the rutting resistance of asphalt mixture in the HWTT by the viscoplastic strain of the asphalt mixture itself under repeated loading.

**Figure 7:**
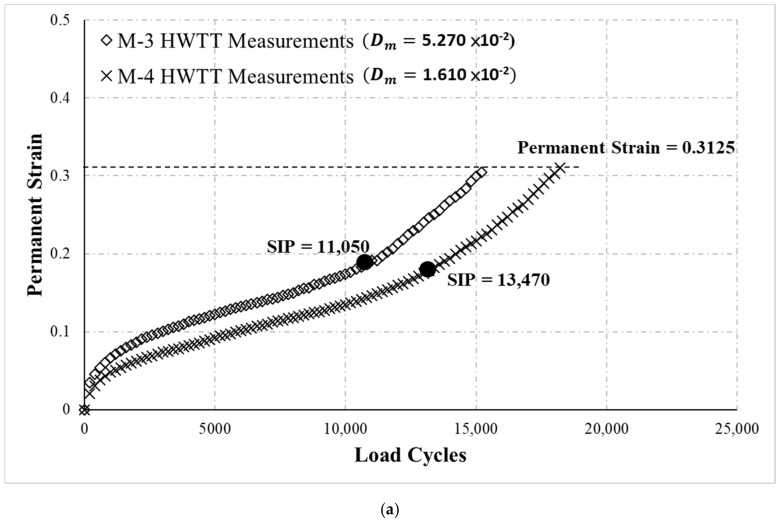

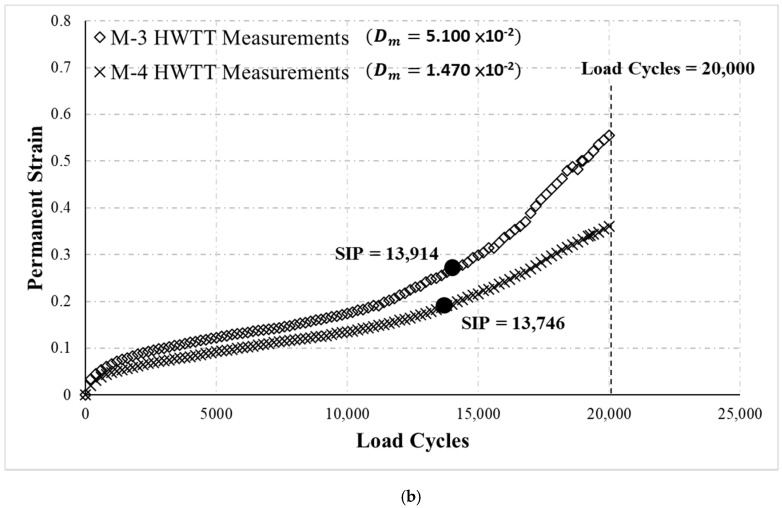
HWTT results for M-3 and M-4. (**a**) With a rut depth of 12.5 mm as the ending point of the data analysis. (**b**) With 20,000 load cycles as the ending point of the data analysis.

Figure 7 provides the HWTT results for the M-3 and M-4 specimens. During the test, the specimens were continually loaded for 20,000 load cycles regardless of whether the rut depth reached 12.5 mm. The results were organized in two ways: (i) two sets of test data were extracted until the accumulated rut depth was 12.5 mm (permanent strain = 0.3125), as indicated in Figure 7a, and (ii) two sets of test data were extracted at 20,000 load cycles (Figure 7b). Subsequently, the SIP of the two datasets was calculated following HWTT specifications. As Figure 7a reveals, the SIP of M-3 and M-4 was 11,050 and 13,470, respectively, when the ending point was at an accumulated rut depth of 12.5 mm. The results concluded that M-4 was more resistant to moisture damage than was M-3. However, with an ending point of 20,000 load cycles, the SIP of M-3 and M-4 was 13,914 and 13,746, respectively (Figure 7b). This result was contrary to the moisture susceptibility evaluated using an accumulated rut depth of 12.5 mm as the data interval. With the proposed method to calculate the moisture susceptibility indicator $D_{m}$, when the data ending point was a rut depth of 12.5 mm, the $D_{m}$ of M-3 and M-4 was 5.270 × 10^−2^ and 1.610 × 10^−2^, respectively (Figure 7a). When the test data were analyzed with an ending point of 20,000 load cycles, the $D_{m}$ of M-3 and M-4 was 5.100 × 10^−2^ and 1.470 × 10^−2^, respectively. According to these results, the proposed indicator $D_{m}$ in groups (i) and (ii) demonstrates that M-3 was more susceptible to moisture damage, and the $D_{m}$ from data analysis of the groups did not substantially differ. This result suggests that the proposed $D_{m}$ in this study was not significantly affected by the ending point of data analysis or the range of data analyzed. Hence, the proposed novel method and $D_{m}$ can more effectively evaluate the moisture susceptibility of asphalt mixtures in the HWTT.

The analysis of moisture susceptibility of M-3 and M-4 was further conducted using the method proposed by Yin et al. [20]. The analysis also determined the ${\mathrm{LC}}_{\mathrm{SN}}$, the loading number at which the moisture damage begins to affect the asphalt mixture in the HWTT, like the proposed $N_{\mathrm{SN}}$ in this study. Then, the moisture susceptibility index ${\mathrm{LC}}_{\mathrm{ST}}$ was defined as the number of additional load cycles needed for the rut depth accumulated to reach 12.5 mm after ${\mathrm{LC}}_{\mathrm{SN}}$ in the HWTT [20]. The results of SIP, $D_{m}$, and ${\mathrm{LC}}_{\mathrm{ST}}$ were summarized in Table 2. When the ending point was at a rut depth of 12.5 mm, the ${\mathrm{LC}}_{\mathrm{ST}}$ was 13,915 and 16,723 for M-3 and M-4, respectively. With the ending point of 20,000 load cycles, the ${\mathrm{LC}}_{\mathrm{ST}}$ was 14,996 for M-3 and 17,285 for M-4. The results demonstrate that M-4 was more resistant to moisture damage in both groups (i) and (ii), which was consistent with the proposed $D_{m}$ results. According to the results of $D_{m}$ and ${\mathrm{LC}}_{\mathrm{ST}}$, both indicators were not significantly affected by the ending point of data analysis, revealing that the determination of the loading cycle at which moisture damage to the asphalt mixture begins in the HWTT is essential for evaluating the moisture susceptibility of asphalt mixtures.

**Figure 8:**
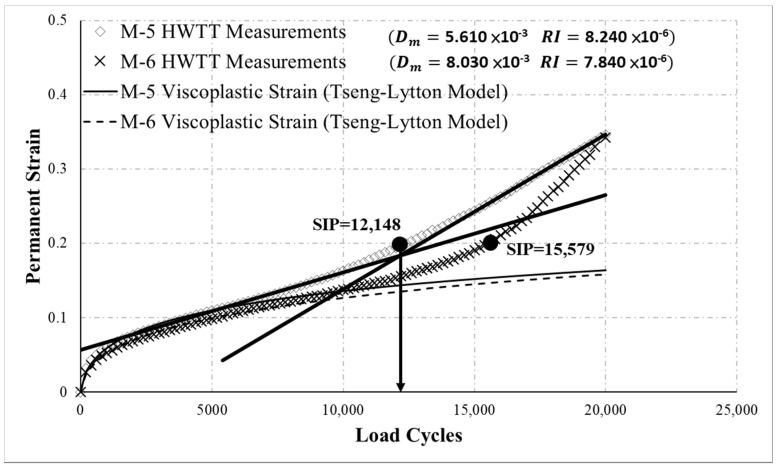
HWTT results for M-5 and M-6.

Figure 8 presents the HWTT results for M-5 and M-6. The curves in the diagram indicate that M-6 entered the damage phase before 20,000 load cycles, as evidenced by the moisture damage. By contrast, M-5 was not as susceptible to moisture damage; however, the SIP calculated following the HWTT specifications was 12,148 for M-5 and 15,579 for M-6, suggesting that M-6 was more resistant to moisture damage, which was contrary to the curve trend. This result was attributed to a minor change in the damage-phase curve of M-5, which produced a flatter slope when a straight line was used to fit the damage phase, causing the SIP to shift forward (bold lines in Figure 8). Therefore, using the HWTT specification to analyze asphalt mixture that does not develop a noticeable damage phase can easily lead to an incorrect evaluation of moisture susceptibility. With the proposed moisture susceptibility indicator, $D_{m}$ was 5.610 × 10^−3^ for M-5 and 8.030 × 10^−3^ for M-6, suggesting that M-5 was more resistant to moisture damage than was M-6, consistent with the curve trend. This analysis revealed that the proposed indicator can more closely reflect the moisture susceptibility of asphalt mixtures. In addition, the curves suggest that M-5 and M-6 behaved similarly in the creep phase, and the rutting of M-5 was slightly deeper than that of M-6. The proposed analysis in this study determined that RI was 8.240 × 10^−6^ for M-5 and 7.840 × 10^−6^ for M-6, indicating that M-5 and M-6 had comparable resistance to rutting, with M-6 slightly more resistant. This finding coincides with the curve trend. However, if the conventional HWTT specification was used, then M-6 was more resistant to rutting at 15,000 load cycles, but at 20,000 load cycles M-5 and M-6 had comparable rutting resistance. These results add evidence that the conventional rutting resistance in HWTT specification are clearly affected by the number of load cycles selected.

**Figure 9:**
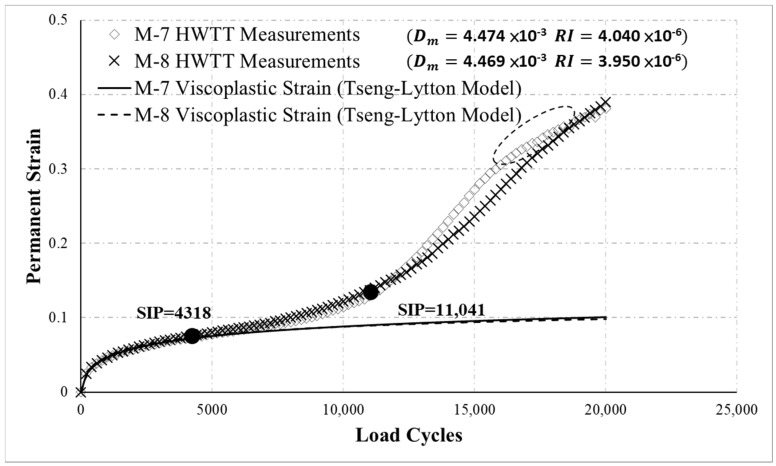
HWTT results for M-7 and M-8.

Figure 9 presents the HWTT results for specimens M-7 and M-8 asphalt mixtures. In the figure, the development trend of the curves signifies that M-7 and M-8 accumulated permanent strain in a similar manner. According to the results obtained using the proposed method, $D_{m}$ was 4.474 × 10^−3^ for M-7 and 4.469 × 10^−3^ for M-8, indicating that M-7 and M-8 exhibited comparable susceptibility to moisture damage, which was consistent with the curve trend. However, analysis of moisture susceptibility using HWTT determined that the SIP of M-7 and M-8 was 4318 and 11,041, respectively, suggesting that M-8 was considerably more resistant to moisture damage. This inconsistency was attributed to unstable data on asphalt mixture in the damage phase. The end of the M-7 curve (dashed line with circles in the figure) signifies that the permanent strain of M-7 increased slowly at the end. This behavior differs from the failure behavior of the asphalt mixture (the rate of permanent strain should increase in the damage phase). The SIP in the HWTT is the point of intersection of the regression lines of the creep and damage phases. Therefore, instability in the damage phase data often causes bias in the regression straight line of this phase (the bold lines in Figure 9), leading to incorrect results regarding the SIP and impeding the evaluation of moisture susceptibility. The proposed indicator of moisture susceptibility was not affected by instability in the data at the damage phase and can effectively determine the moisture susceptibility of asphalt mixture. Regarding rutting resistance, the RI of M-7 and M-8 were 4.040 × 10^−6^ and 3.950 × 10^−6^, respectively, indicating similar rutting resistance. This result coincided with the trend of the curves.

**Figure 10:**
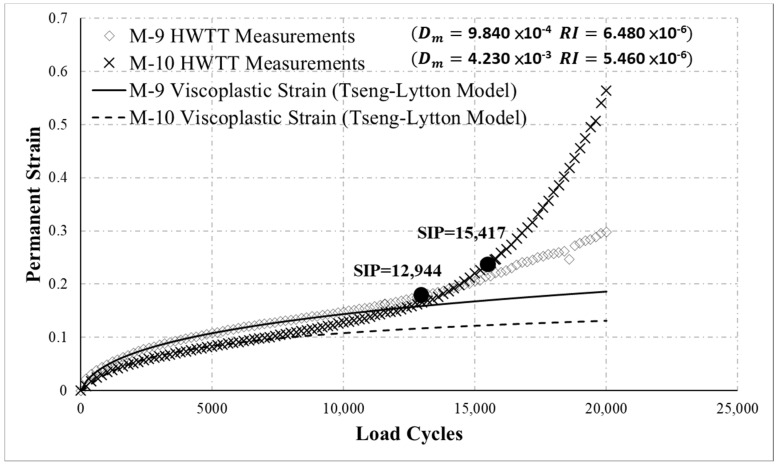
HWTT results for M-9 and M-10.

Figure 10 displays the HWTT results for M-9 and M-10. The curves in the diagram indicate M-9 was more resistant to moisture damage than was M-10 (the permanent strain in the last portion of the M-10 curve increase considerably faster). However, an analysis of the SIP based on the HWTT specification indicated that M-10 had more considerable resistance to moisture than M-9 (the SIP was 12,944 for M-9 and 15,417 for M-10). This inconsistency was attributed to an exponential increase in the M-10 curve in the damage phase; therefore, a straight line cannot effectively fit the test data of this phase (the bold lines in Figure 10), rendering the SIP undeterminable. The results of the new $D_{m}$ indicator were 9.840 × 10^−4^ for M-9 and 4.230 × 10^−3^ for M-10, revealing that M-9 was more resistant to moisture damage. This result clearly demonstrates that the $D_{m}$ can effectively characterize the moisture susceptibility of asphalt mixtures. In addition, with the proposed RI, M-9 and M-10 had a resistance of 6.480 × 10^−6^ and 5.460 × 10^−6^ to rutting, respectively. This indicates that M-10 was more resistant to rutting, consistent with the trends of the curves. The rutting resistance in the HWTT suggested that M-10 was not as resistant to rutting (e.g., rut depth at 20,000 load cycles) as M-9 was. However, the permanent strain at 20,000 load cycles includes the viscoplastic strain of the material itself and permanent strain due to moisture damage. Since M-10 was more susceptible to moisture damage, permanent strain induced by moisture damage increased dramatically at the later stage of the test, which resulted in a final rut depth greater than that of M-9, resulting in incorrect results regarding rutting resistance.

**Figure 11:**
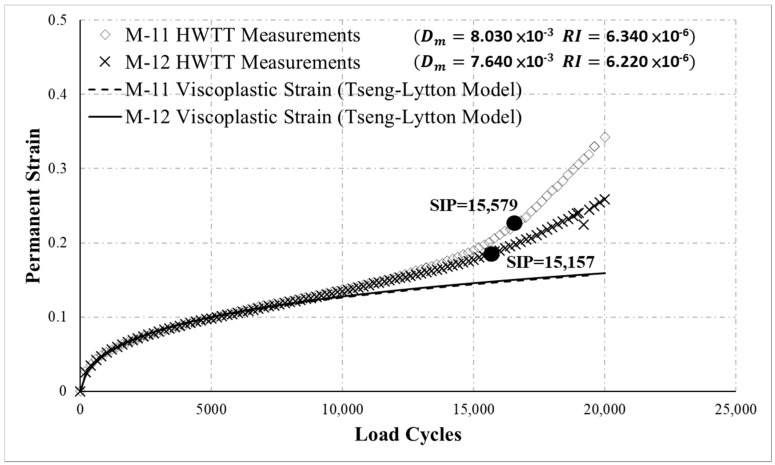
HWTT results for M-11 and M-12.

Figure 11 presents the HWTT results for M-11 and M-12. According to the test results, M-12 had stronger resistance to moisture damage than did M-11, and the proposed moisture susceptibility indicator also determined that M-12 had stronger resistance to moisture damage, as shown in Figure 11. This result was consistent with the trends of the HWTT curves. However, the results for the SIP based on the HWTT specification indicated that M-11 was more resistant to moisture damage. Regarding rutting, the results revealed that the specimens exhibited similar permanent strain behavior in the post-composition and creep phases. This phenomenon suggests that before moisture damage occurred, the specimens had comparable resistance to rutting. The proposed rutting indicator also determined that M-11 and M-12 had comparable resistance to rutting, as illustrated in Figure 11. However, based on permanent strain at a particular number of load cycles (e.g., at 20,000 load cycles), M-11 had weaker resistance to rutting. This result further verified the reliability of the rutting resistance and moisture susceptibility indicators proposed in this study.

**Figure 12:**
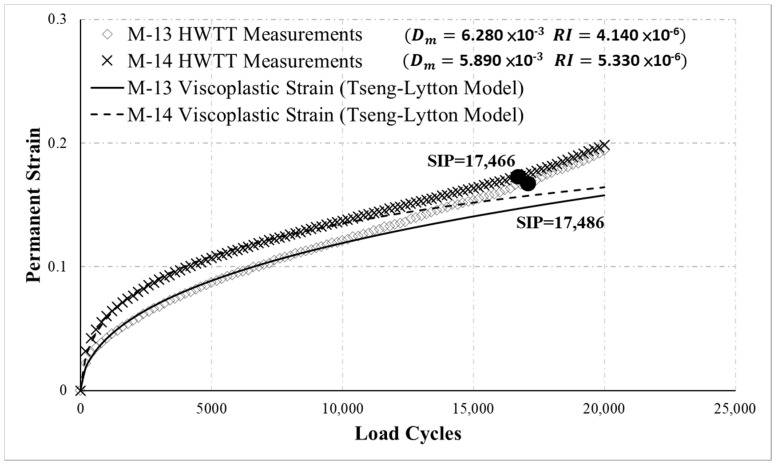
HWTT results for M-13 and M-14.

Figure 12 displays the HWTT curves for M-13 and M-14. The curves indicate that M-13 and M-14 accumulated permanent strain differently. Nevertheless, M-13 and M-14 had similar rut depths during the final 20,000 load cycles. Based on this rut depth, the specimens had comparable resistance to rutting; however, the novel RI revealed that M-13 was more resistant to rutting (RI was 4.140 × 10^−6^ for M-13 and 5.330 × 10^−6^ for M-14). This result was consistent with the curve trends. In sum, the new RI can effectively evaluate the behavior of asphalt mixtures accumulating permanent strain under repeated loading.

## 6. Conclusions and Recommendations

The HWTT is widely used in laboratories to evaluate the rutting resistance and moisture susceptibility of asphalt mixtures; however, the indicators of rutting resistance (rut depth at a particular number of load cycles) and moisture susceptibility (number of load cycles at SIP) defined by the HWTT specifications cannot consistently and effectively evaluate the behaviors of asphalt mixtures in the HWTT. This study established a systematic method of analysis for the HWTT and introduced two new indicators, $D_{m}$ and RI, which were then used to evaluate the rutting resistance and moisture susceptibility of asphalt mixtures. The novel method was used to analyze the HWTT results of 14 field core asphalt mixture specimens with different behaviors. The objective was to apply $D_{m}$ and RI to assess the rutting resistance and moisture susceptibility performance of asphalt mixtures. The proposed indicators were then compared with the conventional HWTT indicators. The conclusions of the study and recommendations for future research are provided as follows:

1. In the HWTT, different mechanical behaviors and mechanisms are involved in the accumulation of permanent strain in various phases. This study used the Francken model and the three-stage model to propose a systematic analytic method that calculates the critical loading number of asphalt mixtures (i.e., $N_{\mathrm{SN}}$, $N_{P-C}$ and $N_{C-D}$) at the intersection of each phase in the HWTT. It also proposed that the new rutting resistance index RI be the rate of change in permanent strain between $N_{P-C}$ and $N_{\mathrm{SN}}$. According to the results of the analysis, when the conventional HWTT indicator of rutting resistance was used to evaluate asphalt mixtures with different rutting accumulation behaviors, the selected number of load cycles may generate completely opposite results. Therefore, the conventional HWTT indicator is greatly affected by the number of load cycles selected. By contrast, the new indicator (RI) can effectively characterize the rutting resistance of asphalt mixtures.
2. The SIP, which is a moisture susceptibility indicator in the HWTT, is defined as the number of load cycles at the point of intersection of straight lines fitted for the creep and damage phases. Therefore, the determination of the SIP is strongly affected by the data for these two phases. According to the analysis, using different data ranges (e.g., 20,000 load cycles or maximum rut depth of 12.5 mm) in the analysis yields different SIP results regarding moisture susceptibility. Furthermore, the effectiveness of the SIP is highly dependent on the data conditions of the damage phase. For instance, minor changes in the damage-phase curve, dramatic changes in damage-phase data, or unreasonable curves often render SIP ineffective for evaluating the moisture susceptibility of asphalt mixtures. This study proposed a novel indicator of moisture susceptibility, $D_{m}$, to be the effect of moisture damage at unit load between $N_{\mathrm{SN}}$ and $N_{C-D}$. The analysis determined that the indicator${\;D}_{m}$ can effectively describe the moisture susceptibility of asphalt mixtures and is relatively stable.
3. The advantage of the novel indicator RI is that it performs analysis on the entire range of data between $N_{P-C}$ and $N_{\mathrm{SN}}$, unlike the current HWTT protocol, which uses rut depth at a particular number of load cycles (single-point data) as the indicator of rutting resistance. In addition, the proposed RI also excludes the effects of initial porosity on rutting resistance. The novel indicator $D_{m}$ performs analysis on the entire curve from $N_{\mathrm{SN}}$ to $N_{C-D}$, unlike the current HWTT, which defines the SIP as the number of load cycles (single-point data). Moreover, $D_{m}$ is based on the area of difference between the HWTT curves of permanent strain (permanent strain due to repeated loading on the asphalt mixture and permanent strain due to moisture damage) and viscoplastic strain (permanent strain due to repeated loading on the asphalt mixture). Therefore, $D_{m}$ can determine based on the effect only by moisture damage and can more effectively evaluate the moisture susceptibility of the asphalt mixture. The proposed indicators of rutting resistance and moisture susceptibility can provide an overall evaluation of the asphalt mixture behaviors based on the curve development in each phase. By contrast, the current HWTT protocol adopts single-point determinations, which cannot provide an overall evaluation of asphalt mixture behaviors and can easily generate erroneous results.
4. The novel indicators RI and $D_{m}$ must be compared with field performance observations to further verify their reliability. In addition, the new analytic method and indicators require further verification with more HWTT results.
5. Field core specimens were analyzed in this study. Future researchers could prepare specimens in the laboratory, where properties relevant to the performance of asphalt mixtures (e.g., porosity, aggregate gradation, asphalt binder grade) can be controlled to further verify the method and indicators introduced in this study.

## Figures and Tables

**Table 1:** Rutting at different numbers of load cycles in the HWTT, RI, and $\Delta \varepsilon_{10,000}^{vp}$ results.

Mixture	Permanent Strain at Certain Number of Load Cycles				RI	$\Delta \varepsilon_{10,000}^{vp}$
	5000	10,000	15,000	20,000		
M-1	0.132	0.191	0.258	0.328	1.01 × 10^−5^	7.82 × 10^−6^
M-2	0.102	0.156	0.300	0.580	8.11 × 10^−6^	4.57 × 10^−6^

**Table 2:** The results of SIP, $D_{m}$ and${\;\mathrm{LC}}_{\mathrm{ST}}$.

Mixture	12.5 mm (Ending Point)			20,000 Cycles (Ending Point)		
	SIP (Cycles)	$D_{m}$	${\mathrm{LC}}_{\mathrm{ST}}\;(\mathrm{Cycles})$	SIP (Cycles)	$D_{m}$	${\mathrm{LC}}_{\mathrm{ST}}\;(\mathrm{Cycles})$
M-3	11,050	5.270 × 10^−2^	13,915	13,914	5.100 × 10^−2^	14,996
M-4	13,470	1.610 × 10^−2^	16,723	13,746	1.470 × 10^−2^	17,285

## References

[1] Romero P., Stuart K. (1998). Evaluating Accelerated Rut Testers.

[2] AASHTO.324-17 (2017). Standard Method of Test for Hamburg Wheel-Track Testing of Compacted Asphalt Mixtures.

[3] Han J., Shiwakoti H. (2016). Wheel tracking methods to evaluate moisture sensitivity of hot-mix asphalt mixtures. Front. Struct. Civ. Eng..

[4] Stuart K.D., Izzo R.P. (1995). Correlation of Superpave G*/sin Delta with Rutting Susceptibility from Laboratory Mixture Tests. Transp. Res. Rec..

[5] Aschenbrener T. (1995). Evaluation of Hamburg wheel-tracking device to predict moisture damage in hot-mix asphalt. Transp. Res. Rec..

[6] Izzo R.P. (1999). Use of the Hamburg Wheel-Tracking Device for Evaluating Moisture Susceptibility of Hot-Mix Asphalt. Transp. Res. Rec..

[7] Kim S., Shen J., Myung Jeong M. Evaluation of Aggregate Sizes on Performance of Georgia Asphalt Mixture Evaluated by Hamburg Wheel Tracking Device. Proceedings of the TRB 96th Annual Meeting, Transportation Research Board.

[8] Rahman F., Hossain M. (2014). Review and Analysis of Hamburg Wheel Tracking Device Test Data.

[9] Shen J., Kim S., Myung Jeong M. (2017). Evaluation of Georgia Asphalt Mixture Properties Using a Hamburg Wheel Tracking Device.

[10] Walubita L.F., Zhang J., Das G., Mushota C., Alvarez A.E., Scullion T. (2012). Hot-mix asphalt permanent deformation evaluated by Hamburg wheel tracking, dynamic modulus, and repeated load tests. Transp. Res. Rec. J. Transp. Res. Board.

[11] Uppu K.K., Hossain M., Ingram L.S., Kreider R. Moisture Susceptibility of Superpave Mixtures with Varying Binder Contents. Proceedings of the Airfield and Highway Pavements.

[12] Kim S., Shen J., Myung Jeong M. (2018). Effects of Aggregate Size on the Rutting and Stripping Resistance of Recycled Asphalt Mixtures. J. Mater. Civ. Eng..

[13] Larrain M.M.M., Tarefder R.A. Rutting Potential of Parameters Using Hamburg Wheel Tracking Device (HWTD). Proceedings of the International Airfield and Highway Pavements Conference.

[14] Larrain M.M.M., Tarefder R.A. Effects of Asphalt Concrete Gradation, Air Voids, and Test Temperatures on Rutting Susceptibility by Using the Hamburg Wheel Tracking Device (HWTD). Proceedings of the Fourth Geo-China International Conference.

[15] Schram S., Williams R.C. Ranking of HMA Moisture Sensitivity Tests in Iowa. Proceedings of the 92nd Annual Meeting of the Transportation Research Board.

[16] Walubita L.F., Faruk A.M., Zhang J., Komba J.J., Alrashydah E.I., Simate G.S. (2019). The Hamburg Rutting Test (HWTT) alternative data analysis methods and HMA screening criteria. Int. J. Pavement Res. Technol..

[17] Al-Khateeb G.G., Basheer I.A. (2009). A Three-Stage Rutting Model Utilizing Rutting Performance Data from the Hamburg Wheel-Tracking Device (WTD). Road Transp. Res..

[18] Tsai B.-W., Harvey J.T., Monismith C.L. (2005). Using the Three-Stage Weibull Equation and Tree-Based Model to Characterize the Mix Fatigue Damage Process. Transp. Res. Rec..

[19] Tsai B.-W., Coleri E., Harvey J.T., Monismith C.L. (2016). Evaluation of AASHTO T 324 Hamburg-Wheel Track Device test. Constr. Build. Mater..

[20] Yin F., Arambula E., Lytton R., Martin A.E., Cucalon L.G. (2014). Novel Method for Moisture Susceptibility and Rutting Evaluation Using Hamburg Wheel Tracking Test. Transp. Res. Rec. J. Transp. Res. Board.

[21] Schram S., Williams R.C., Buss A. (2014). Reporting Results from the Hamburg Wheel Tracking Device. Transp. Res. Rec. J. Transp. Res. Board.

[22] Zhou F., Scullion T., Sun L. (2004). Verification and Modeling of Three-Stage Permanent Deformation Behavior of Asphalt Mixes. J. Transp. Eng..

[23] Francken L., Vanelstraete A., Verhasselt A. Long term ageing of pure and modified bitumen: Influence on the rheological properties and relation with the mechanical performance of asphalt mixtures. Proceedings of the Eight International Conference on Asphalt Pavements.

[24] Chaturabong P., Bahia H. (2017). The evaluation of relative effect of moisture in Hamburg wheel tracking test. Constr. Build. Mater..

[25] Tseng T.-H., Lytton R.L. (1989). Prediction of Permanent Deformation in Flexible Pavement Materials. Implication of Aggregates in the Design, Construction, and Performance of Flexible Pavements.

